# Next-generation DNA sequencing identifies novel gene variants and pathways involved in specific language impairment

**DOI:** 10.1038/srep46105

**Published:** 2017-04-25

**Authors:** Xiaowei Sylvia Chen, Rose H. Reader, Alexander Hoischen, Joris A. Veltman, Nuala H. Simpson, Clyde Francks, Dianne F. Newbury, Simon E. Fisher

**Affiliations:** 1 Language and Genetics Department, Max Planck Institute for Psycholinguistics, Nijmegen, The Netherlands; 2 Wellcome Trust Centre for Human Genetics, University of Oxford, Oxford, OX3 7BN, UK; 3 Department of Human Genetics, Radboud University Medical Center, Nijmegen, The Netherlands; 4 Department of Clinical Genetics, University of Maastricht, Maastricht, The Netherlands; 5 Donders Institute for Brain, Cognition and Behaviour, Nijmegen, The Netherlands; 6 Department of Biological and Medical Sciences, Faculty of Health and Life Sciences, Oxford Brookes University, Oxford, UK

## Abstract

A significant proportion of children have unexplained problems acquiring proficient linguistic skills despite adequate intelligence and opportunity. Developmental language disorders are highly heritable with substantial societal impact. Molecular studies have begun to identify candidate loci, but much of the underlying genetic architecture remains undetermined. We performed whole-exome sequencing of 43 unrelated probands affected by severe specific language impairment, followed by independent validations with Sanger sequencing, and analyses of segregation patterns in parents and siblings, to shed new light on aetiology. By first focusing on a pre-defined set of known candidates from the literature, we identified potentially pathogenic variants in genes already implicated in diverse language-related syndromes, including *ERC1, GRIN2A*, and *SRPX2*. Complementary analyses suggested novel putative candidates carrying validated variants which were predicted to have functional effects, such as *OXR1, SCN9A* and *KMT2D*. We also searched for potential “multiple-hit” cases; one proband carried a rare *AUTS2* variant in combination with a rare inherited haplotype affecting *STARD9*, while another carried a novel nonsynonymous variant in *SEMA6D* together with a rare stop-gain in *SYNPR*. On broadening scope to all rare and novel variants throughout the exomes, we identified biological themes that were enriched for such variants, including microtubule transport and cytoskeletal regulation.

Developmental disorders of speech and language affect approximately 10% of children at school entry^1^ and are related to educational, behavioural and psychological outcomes. Two primary language-related disorders that have been extensively investigated at the genetic level are specific language impairment (SLI) and developmental dyslexia. They impair spoken and written language skills respectively and are clinically defined as disorders affecting the given domain despite full access to education and no pre-existing neurological disabilities that might explain the impairment, such as an auditory or intellectual deficit^2^. SLI and dyslexia are both highly heritable^3^, and show high comorbidity, with complex genetic underpinnings involving multiple susceptibility loci^4^. However, little is currently known regarding the crucial biological risk mechanisms.

A range of methods have been used to investigate the genetic architecture underlying speech and language disorders. Initial linkage studies of family-based samples identified SLI susceptibility loci on chromosomes 2p22, 10q23^5^, 13q21 (SLI3, OMIM%607134)^6^, 13q33^5^, 16q23–24 (SLI1, OMIM%606711)^7^, and 19q13 (SLI2, OMIM%606712)^7^. Similarly, early studies of families affected by dyslexia uncovered regions of linkage on multiple chromosomes, including 15q21 (DYX1, OMIM#127700)^8^, 6p22.3-p21.3 (DYX2, OMIM%600202)^9^, 2p16-p15 (DYX3, OMIM%604254)^10^, 3p12-q13 (DYX5, OMIM%606896)^11^, 18p11.2 (DYX6, OMIM%606616)^12^, 11p15.5 (DYX7)^13^, 1p36-p34 (DYX8, OMIM%608995)^14^ and Xq27.2-q28 (DYX9, OMIM%300509)^15^. Subsequent investigations have identified associations and/or aetiological chromosomal rearrangements that implicate genes within several of these linkage regions (reviewed by ref. 16). Key genes include *CMIP* (C-maf-inducing protein, OMIM*610112) and *ATP2C2* (ATPase, Ca2+-transporting, type 2c, member 2, OMIM*613082) in SLI1^17^; *DYX1C1* (OMIM*608706) in DYX1^18^; *KIAA0319* (OMIM*609269) and *DCDC2* (Doublecortin domain-containing protein 2, OMIM*605755) in DYX2^19^,^20^,^21^; *C2orf3*/*MRPL19* (Mitochondrial ribosomal protein L19, OMIM*611832) in DYX3^22^; and *ROBO1* (Roundabout, Drosophila, homologue of, 1, OMIM*602430) in DYX5^23^. Additional risk loci and variations are beginning to be suggested by genome-wide association scans (GWAS, reviewed by ref. 24), but few have exceeded accepted thresholds for significance, and they have yet to be validated by independent replication studies.

Although the majority of speech and language impairments are modeled as complex genetic disorders, there is increasing evidence that common DNA variations are unlikely to provide a full account of their molecular basis^24^. Thus, although linkage and association studies have identified strong evidence of a genetic influence, many rarer variants with aetiological relevance may be overlooked because they will not be captured by single nucleotide polymorphism (SNP) arrays, or do not reach stringent significance parameters. Recent findings indicate that the boundary between common traits and monogenic forms of disorder may be less defined than previously thought^25^,^26^. Accordingly, with advances in molecular technologies, examples can be drawn from the literature of rare or private high-penetrance variants that contribute to certain forms of speech and language deficits^24^. Mutations of the *FOXP2* transcription factor (Forkhead box, P2, OMIM*605317) are known to lead to developmental syndromes involving verbal dyspraxia, or childhood apraxia of speech, accompanied by problems with many aspects of language^27^,^28^. *FOXP1* (Forkhead box P1, OMIM*605515), a paralogue of *FOXP2*, has similarly been implicated in neurodevelopmental disorder^29^,^30^, along with some of its transcriptional targets, most notably, *CNTNAP2* (Contactin-associated protein-like 2, OMIM*604569)^31^,^32^. Rare variants of the *FOXP2* target *SRPX2* (Sushi-repeat-containing protein, X-linked, 2, OMIM*300642)^33^ have been identified in epileptic aphasias^34^, as have mutations of *GRIN2A* (Glutamate receptor, ionotropic, N-methyl-D-aspartate, subunit 2A, OMIM*138253)^35^,^36^,^37^. Moreover, the closely related gene *GRIN2B* (Glutamate receptor, ionotropic, N-methyl-D-aspartate, subunit 2B, OMIM*138252) has also been implicated in language-relevant cognitive disorders^38^,^39^,^40^. Overlaps between rare deletions and duplications that yield speech, language and/or reading disruptions have highlighted several additional candidate genes; including *ERC1* (ELKS/RAB6-interacting/CAST family member 1), *SETBP1* (SET-binding protein 1, OMIM*611060), *CNTNAP5* (Contactin-associated protein-like-5, OMIM*610519), *DOCK4* (Dedicator of cytokinesis 4, OMIM*607679), *SEMA6D* (Semaphorin 6D, OMIM*609295), and *AUTS2* (Autism susceptibility candidate 2)^41^,^42^,^43^,^44^,^45^,^46^,^47^. Most recently, studies of geographically isolated populations have identified coding variants that have been postulated to contribute to speech and language difficulties in these populations^48^,^49^. Overall, this body of work points to the importance of rare and/or private variants in language-related phenotypes, suggesting that high-resolution molecular technologies like next-generation DNA sequencing hold considerable promise for unraveling a disorder such as SLI.

Thus, in this study, we performed exome sequencing of 43 probands affected by severe language impairment without a known cause. We employed complementary hypothesis-driven approaches to identify putative aetiological variants and associated biological processes. Our investigation detected cases with potential pathogenic mutations, and highlighted molecular pathways that may be important to speech and language development.

## Results

### Exome sequencing in SLI

We performed whole exome sequencing of 43 unrelated probands affected by SLI (see *Methods*). On average, 129.3 million mapped reads (median = 133.3; min = 67.1; max = 173.3) were generated per sample. Across all 43 samples, an average of 85.5% of the target sequence was captured at a minimum read depth of ten. The mean read depth of the exonic regions was 86.8, with 39.5% of reads reaching this level. Sequence metrics can be found in Supplementary Table S1. The coverage versus read depth of all samples is shown in Supplementary Figure S1.

In total, across all 43 probands, 353,686 raw variant calls were made, of which 62.2% fell outside known coding sequence. After removing variants with low quality (see *Methods*), 270,104 remained. 35,550 (13.2%) of these were predicted to affect protein coding, including 34,571 nonsynonymous variants, 549 stop-gains/losses, and 430 splice-site variants. On average there were 8,594 (range 7,655–10,380) nonsynonymous variants, 91 (65–114) stop-gains/losses, and 72 (50–98) splice-site variants per individual (Supplementary Table S2).

The transition versus transversion ratio (Ti/Tv) for all SNVs within the exonic regions was 2.81, higher than the value observed for all variants (Supplementary Table S1), and in line with that expected^50^. The total variants corresponded to 48,722 variants per individual (min = 43,699; max = 58,260) (Supplementary Table S1), the majority of which were common SNPs seen across all probands. As part of a prior published study^51^, all 43 samples had previously been genotyped on Illumina HumanOmniExpress-12v1Beadchip (San Diego, CA, USA) arrays, which include ~750,000 common SNPs. 40,267 variants identified by our exome sequencing had been directly genotyped on the arrays and for these common SNPs, we observed a genotype concordance rate of 97%. The numbers of rare and novel variants identified per individual are shown in Supplementary Table S3.

In the first stage of analysis, we performed a tightly constrained search for aetiologically relevant variants, using several complementary methods. We began by identifying all variants occurring within a selection of known candidate genes that have previously been suggested as susceptibility factors in primary speech, language and/or reading disorders. Next, we characterized rare or novel variants of potential high risk from elsewhere in the exome by defining stop-gain variants, as well as searching for potential cases of compound heterozygotes for rare disruptive variants. Finally, we looked for likely “multiple-hit” events by searching for probands who carried more than one event of potential significance across different genes. For all variants in this stage of analysis we performed independent validation using Sanger sequencing, and assessed inheritance patterns in the available siblings and parents. Given the relatively small sample size of our study, these constraints provide a framework to maximize our chances of identifying contributory variants under an assumption that those variants will explain a large proportion of the trait variance. Throughout this paper, we refer to guidelines for inferring likely causality, as proposed by MacArthur and colleagues^52^.

In the second stage of analysis, we broadened our scope to consider all rare and novel variants identified throughout the exome, and tested for biological pathways that showed enrichment in our dataset, using within-proband and group-based approaches. Moreover, we assessed how the pattern of findings was affected by the relative frequency of the variants being studied. Thus, this second stage went beyond the level of individual genes to provide a foundation for exploring potential mechanisms that could be involved in aetiology of SLI.

### Nonsynonymous variants in selected candidate genes

According to current guidelines for evaluating causality in whole exome/genome datasets, genes previously implicated in similar phenotypes should be evaluated before exploring potential new candidates^52^. Therefore, prior to beginning any bioinformatic analyses of our exome data, we performed a literature search to identify a set of candidate genes that had been most reliably implicated in speech, language and reading disorders by earlier research. This literature survey yielded 19 candidate genes: *CMIP, ATP2C2, CNTNAP2* and *NFXL1*, which have previously been associated with common forms of SLI^17^,^31^,^48^; *FOXP2*, which is involved in a monogenic form of speech and language disorder^27^,^28^, and its orthologue *FOXP1*, which has also been implicated in relevant neurodevelopmental disorders^29^; *DYX1C1, KIAA0319, DCDC2*, and *ROBO1*, which are candidate genes in developmental dyslexia^53^; *SRPX2* and *GRIN2A*, which have been implicated in speech apraxia and epileptic aphasias^34^,^36^, as well as the closely related candidate *GRIN2B*^39^,^40^; and, *ERC1, SETBP1, CNTNAP5, DOCK4, SEMA6D*, and *AUTS2*, each of which has been shown to have rare deletions or translocations that yield speech, language and/or reading disruptions^41^,^42^,^43^,^44^,^45^,^46^,^47^.

We identified 37 coding or splice-site variants (36 SNVs, 1 insertion), that were successfully validated by Sanger sequencing, found in 14 of the 19 candidate genes (Table 1). A full list of these candidate-gene variants can be found in Supplementary Table S4. Seventy percent of validated calls represented common variants (population allele frequencies of >1% in 1000 Genomes), 16.2% were rare variants (population frequencies <1% in 1000 Genomes) and 13.5% represented novel changes (not present in 1000 Genomes or EVS) (Table 1).

**Figure 1:**
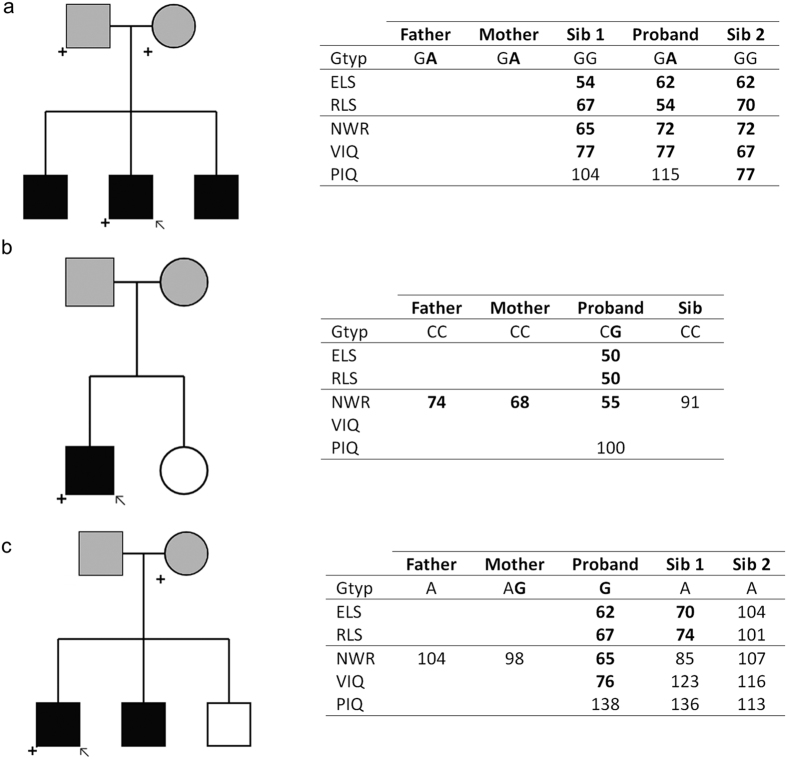
Variants of putative significance in candidate genes. (**a**) *ERC1*, Proband 23. Chr12:1137072, NM_178039:exon2:c.G3A:p.M1I (start-loss). Both parents report history of speech and language problems. All children have special educational needs. (**b**) *GRIN2A*, Proband 4. Chr16:9916226, rs77705198, NM_001134407:exon10:c.G2063C:p.G688A (*de novo*). Mother reports history of speech and language problems (although both parents have low NWR scores). Proband has special educational needs. (**c**) *SRPX2*, Proband 41. ChrX:99922289, rs121918363. NM_014467:exon9:c.A980G:p.N327S. Parents do not report history of speech and language problems. All children have special educational needs. Proband is denoted by arrow. Individuals carrying variant allele are denoted by a plus symbol. Affected individuals are shaded black, unaffected are white, unknown are grey. Parents are always shaded as unknown as the language tests employed were for children only. Self-reported family history is given in text. Additional genotypic and phenotypic information is presented in inset table. Variant alleles are shown in bold. Affection status for all children was defined as CELF-R receptive (RLS) or expressive (ELS) language score >1.5 SD below mean (see *Methods* for details). We also present information regarding nonword repetition ability (NWR) and verbal and non-verbal IQ (VIQ and PIQ respectively). Although these additional scores were not used to ascertain affection status, they can provide useful information regarding specific deficits in individuals. NWR is thought to provide an index of phonological short term memory, while the IQ measures indicate a general level of verbal and non-verbal ability. All measures are standardized with a mean of 100 and a SD of 15. Scores >1.5 SD below the mean are shown in bold.

In total, we observed 5 novel variants (in *ERC1, GRIN2A, GRIN2B, CNTNAP2* and *SEMA6D*) and 6 rare SNVs (in *ATP2C2, AUTS2, CNTNAP5, ROBO1* and *SRPX2*) in the predefined set of candidate genes (Table 2). All of these variants led to nonsynonymous changes. Those with an EVS European American allele frequency of <1% (n = 9) were subsequently sequenced in available relatives to examine their segregation within the nuclear families (Fig. 1, Supplementary Figure S2). Three such variants were considered the most likely to represent pathogenic changes based upon their inheritance, position in the protein and findings from previous literature. These include a *de novo* substitution (p.G688A) in a sporadic case in *GRIN2A* (with true *de novo* status validated via SNP data), a start-loss (disruption of the first methionine codon) in *ERC1* and a substitution (p.N327S) in *SRPX2* (Fig. 1). We also observed a novel substitution in *SEMA6D* (p.H807D), and rare nonsynonymous changes in *AUTS2* (p.R117C) and *ROBO1* (p.V234A) that co-segregated with disorder in affected relatives of the respective probands (Supplementary Figure S2).

### Variants of higher risk: rare stop-gains and potential compound heterozygotes

We next extended our investigation beyond known candidate genes, using two strategies to highlight coding variants of potential deleterious effect from elsewhere in the genome. In one approach, we identified and validated stop-gain variants in our dataset which are rare (<1% in EVS and 1000 Genomes) or novel. (We did not detect any validated rare/novel stop-loss or frame-shift variants in this dataset.) Stop-gain variants result in truncated proteins and have potential to yield more severe consequences than the majority of single amino-acid substitutions. In the other approach, we searched for genes that carried more than one rare, disruptive variant in the same proband, which may represent potential compound heterozygotes. (There were no instances where rare/novel disruptive variants occurred in the homozygous state in the cohort.) Within our sample, these approaches allowed us to focus upon variants that carry an increased chance of being deleterious. As recommended by MacArthur and colleagues^52^, we targeted rare and novel variants, drawing upon large, ethnically matched control data and employing multiple bioinformatic prediction algorithms to evaluate potential pathogenicity. Moreover, again following accepted guidelines, we validated all variants of interest with an independent method (Sanger sequencing) and investigated co-segregation patterns within family units^52^.

**Figure 2:**
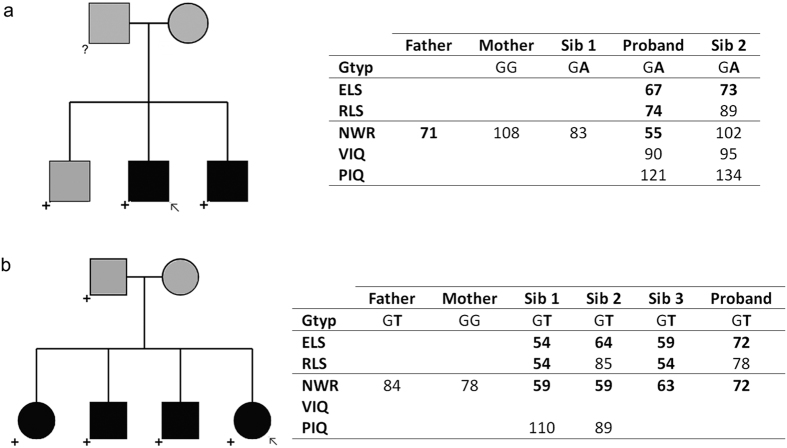
Co-segregating stop-gain variants. (**a**) *OXR1*, Proband 29. Chr8:107738486, rs145739822, NM_001198534:exon1:c.G15A:p.W5X. Father reports history of speech and language problems. No DNA sample was available for father. Proband and sibling 2 have special educational needs. Sibling 1 does not have language or IQ scores available, but has been diagnosed with dyslexia. (**b**) *MUC6*, Proband 8. Chr11:1027390, rs200217410, NM_005961:exon17:c.C2109A:p.C703X. Mother reports history of speech and language problems. Proband has special educational needs. For key for symbols used in this figure, please refer to Fig. 1.

Following annotation and data filtering, we successfully validated 7 rare or novel stop-gain variants. These validated variants were found in the *OR6P1* (Olfactory receptor, family 6, subfamily P, member 1), *NUDT16L1* (Nudix (Nucleoside Diphosphate Linked Moiety X)-Type Motif 16-Like 1), *SYNPR* (Synaptoporin), *OXR1* (Oxidation resistance 1, OMIM*605609), *IDO2* (Indoleamine 2,3-dioxygenase 2, OMIM*612129), *MUC6* (Mucin 6, OMIM*158374) and *OR52B2* (Olfactory Receptor, Family 52, Subfamily B, Member 2) genes. Each was <0.25% in reference samples and found to occur in a heterozygous state in a single proband in our dataset (Table 3). None occurred in known candidate genes for neurodevelopmental disorders. Note that olfactory receptor and mucin family genes are especially susceptible to false positive findings in next-generation sequencing, due to mapping artefacts (http://massgenomics.org/2013/06/ngs-false-positives.html). Thus, although these variants were validated by Sanger sequencing, they should be treated with caution. We again investigated the segregation of these variants within nuclear families (Supplementary Figure S3). Two variants showed evidence of co-segregation with disorder. One validated stop-gain, very near the start of the *OXR1* gene (NM_001198534:p.W5X, NM_001198535:p.W5X), was found in three children from a family, two affected by SLI necessitating special educational needs and a third with a diagnosis of dyslexia (Fig. 2). The variant was not found in the mother, suggesting that it was most likely inherited from the father, who reports a history of speech and language difficulties but for whom we do not have any genetic information. In another pedigree, a validated stop-gain in *MUC6* (NM_005961:p.C703X) was passed from a father to four children, all of whom had expressive and receptive language difficulties (Fig. 2).

In screening for potential cases of compound heterozygotes, we identified 11 genes which carried two or more rare or novel variants in the same proband (Table 4, Supplementary Figure S4). Upon family screening, four such cases were found to represent possible compound heterozygotes where two rare, potentially deleterious variants were inherited from opposite parents and co-segregated with disorder in the children (Supplementary Figure S4). The relevant variants occurred in the *FAT3* (Fat tumor suppressor, Drosophila, homologue of, 3, OMIM*612483), *KMT2D* (Histone-lysine N-methyltransferase 2D, OMIM*602113), *SCN9A* (Sodium channel, voltage-gated, type IX, alpha subunit, OMIM*603415) and *PALB2* (Partner and localizer of *BRCA2*, OMIM*610355) genes. Heterozygous mutations in the *SCN9A* gene have previously been associated with generalized epilepsy with febrile seizures (OMIM#613863) and Dravet syndrome (severe myoclonic epilepsy of infancy, OMIM#607208) when accompanied by mutations in the *SCN1A* (Sodium channel, neuronal type 1, alpha subunit, OMIM*182389) gene^54^,^55^. Loss-of-function mutations in *KMT2D* have been reported to cause Kabuki syndrome (OMIM#147920)^56^,^57^,^58^, a severe syndromic form of intellectual disability associated with dysarthria and oromotor deficits, microcephaly and nystagmus^59^. The *KMT2D* variants in our cohort were rare nonsynonymous changes, rather than confirmed loss-of-function mutations, and the individuals who carried them did not show features of Kabuki syndrome.

### Probands with multiple variants of putative interest

**Figure 3:**
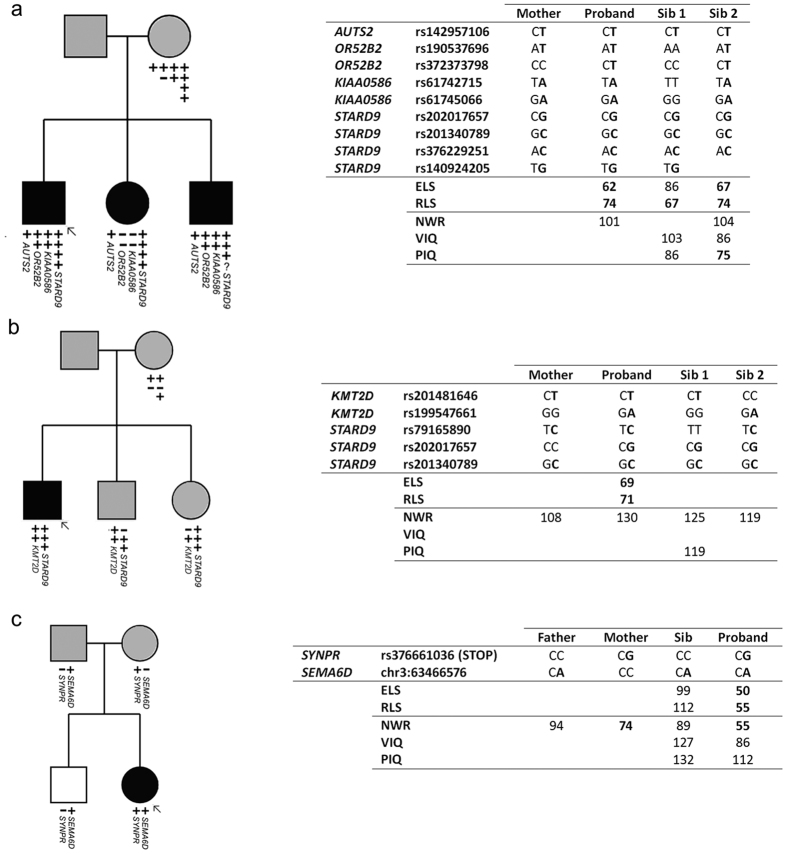
Probands with multiple hits of putative interest. (**a**) Proband 19. Rare *AUTS2* variant, stop and rare variant in *OR52B2*, rare variants in *KIAA0586* and *STARD9*. Parents do not report history of speech and language problems. No sample available for father. All children have special educational needs. (**b**) Proband 12. Multiple rare variants in *KMT2D* and *STARD9*. No family history available but maternal NWR score in normal range. No sample available for father. (**c**) Proband 30. *SYNPR* rare stop variant and *SEMA6D* novel nonsynonymous variant. Parents do not report history of speech and language problems (although mother has low NWR score). Proband has special educational needs. For key for symbols used in this figure, please refer to Fig. 1.

Four of the 43 probands investigated carried more than one rare variant across our prioritized high-risk categories described above, potentially representing “multiple-hit” events. The proband carrying a rare coding variant in *AUTS2* also had a stop-gain in *OR52B2*, and multiple rare variants in each of the *OR52B2, KIAA0586* (OMIM*610178) and *STARD9* (Start domain-containing protein 9, OMIM*614642) genes, all of which were successfully confirmed with Sanger sequencing. The majority of these variants were inherited from a mother who did not report a history of speech and language problems. Both siblings in this family were affected and both carried the rare variants in *AUTS2* and *STARD9* (Fig. 3). Interestingly, in another family, a proband also carried multiple rare validated variants in the *STARD9* gene together with the rare missense variants in *KMT2D* mentioned above (Fig. 3). In both families, the *STARD9* variants were not compound heterozygotes but instead appeared to represent inherited overlapping rare haplotypes that harboured multiple coding variants. One further proband carried a novel nonsynonymous variant in the *SEMA6D* (Semaphorin 6D, OMIM*609295) gene together with a rare stop-gain in the *SYNPR* gene (Fig. 3). The proband is the only family member to inherit both variants and is the only family member with a history of speech and language impairment. Finally, one other family carried a novel variant in *GRIN2B* (Supplementary Figure S2) and two rare coding variants in *MYO19*. However, there was no obvious pattern of co-segregation across these variants.

### Biological function enrichment analysis of genes with rare and novel SNVs

Prior studies suggest that, with a few prominent exceptions^28^, most cases of speech and language impairments follow a complex disorder model where risk is determined by combinations of deleterious variants^60^,^61^. This is further supported by the observation of multiple rare events of potential significance in a subset of our families, described above. We therefore extended our studies to perform an exploratory exome-wide investigation that considered protein interaction pathways and networks. Although our sample is relatively small, these investigations are an important first step towards an unbiased assessment of the role of rare variants in SLI and will help direct further studies in larger sample sets.

Within each proband, we generated a gene set corresponding to transcripts carrying novel or rare (≤1% population frequency) stop-gain, splice-site, or nonsynonymous SNVs that were predicted to be deleterious by SIFT or Polyphen, allowing the investigation of protein-interaction pathways within individuals. Pathways that were significantly shared by more than half of the probands included cell adhesion, regulation of the actin cytoskeleton, calcium signaling and integrin cell-surface interactions (FDR < 0.01, Supplementary Table S5).

We went on to pool these gene sets across all probands (based on a total of 2,818 SNVs, listed in Supplementary Table S6) enabling the identification of gene ontology (GO) classes that were over-represented at the group level with respect to rare SNVs predicted to be deleterious. The most significantly enriched GO term was GO:0001539: “ciliary of bacterial-type flagellar motility” (P = 8.33 × 10^−5^), which is a small functional group consisting of 27 genes (Table 5). Twelve Dynein genes contributed to the 5-fold enrichment in this class. Other significantly over-represented terms included microtubule-based movement, cell adhesion, and actin cytoskeletal organization (FDR < 0.01, Table 5).

**Figure 4:**
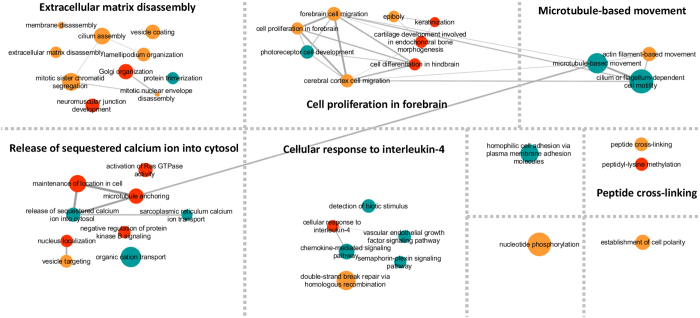
Clusters of significant GO terms enriched with variants of different frequency. Enriched GO terms were identified using three gene lists marked by variant frequency (novel, less than 1%, and between 1–5%). The resulting GO terms associated with the three gene lists are colour-coded (Cyan: between 1–5%; Gold: less than 1%; Red: novel) and with size representing the number of genes within each GO term. The GO terms were clustered based on their functional similarity. Five major functional categories could be identified, namely “Extracellular Matrix Disassembly”, “Cell Proliferation in Forebrain”, “Microtubule-based Movement”, “Release of Sequestered Calcium ion into Cytosol”, and “Cellular response to interleukin-4”. Lines connecting the GO terms indicate levels of similarity between each connected pair.

In a final exploratory step, we investigated the effects of expected variant frequency on pathway representation. These analyses involved a relaxed gene list in which no restrictions were applied in terms of SIFT/polyphen predictions (i.e. all non-synonymous, stop-gain and stop-loss variants with population frequency of ≤5%). The list was split into three discrete segments based on expected frequency; genes which carried novel variants (3,876 variants that were not reported in the 1000 Genomes or EVS, as shown in Supplementary Table S7), genes which carried variants that had been reported in the 1000 Genomes with a variant frequency of <1% (7,084 variants, as shown in Supplementary Table S8) and, an additional set of genes with variants of expected 1000 Genomes frequency between 1% and 5% (4,971 variants, as shown in Supplementary Table S9). Four related themes were found to be significant across variant frequency groups – microtubule-based movement, neuromuscular junction development, cilia and sequestration of calcium ions (Table 6). In general however, significant GO terms were found to cluster differently between frequency classes (Fig. 4). Genes carrying variants in the higher frequency group (1% to 5%) were predominantly localized within the classes “Cellular response to interleukin-4” and “Microtubule-based movement” while the GO enrichments for “Cell proliferation in forebrain” and “Extracellular matrix disassembly” relate mainly to the rarer variants (less than 1% and novel) (Fig. 4).

## Discussion

In this study, we used exome sequencing followed by Sanger validations and segregation analyses, to perform a characterization of exome variants of likely aetiological relevance in SLI, a common form of developmental language disorder. In a dataset of 43 well-phenotyped probands, based on validation, bioinformatics characterization and previous associations, we observed potentially pathogenic variants in several genes that have already been implicated in speech- and language-related syndromes. Specifically, we identified a private start-loss variant in *ERC1*, a gene previously implicated in childhood apraxia of speech^45^; a novel *de novo* substitution disrupting *GRIN2A*, a gene mutated in epilepsy-aphasia spectrum disorders^36^,^62^,^63^; and a hemizygous disruption of *SRPX2* that has previously been identified in people with Rolandic epilepsy with speech apraxia^34^. Thus, although the language difficulties in SLI must (by definition) be unexpected, our findings suggest that a proportion of affected children might actually represent cases of undiagnosed developmental syndromes that may be clinically identifiable. As a note of interest, the three candidate genes highlighted above all show links with epilepsy and/or motor speech problems. Although this may represent a selection bias, it raises the possibility that certain clinical features could be useful endophenotypes for helping to identify high-penetrance coding variants in speech and language disorders.

Consistent with accepted guidelines for defining SLI, none of the probands of our cohort were diagnosed with epilepsy. Yet, two of the three genes noted above were previously implicated in language-related forms of epilepsy. Disruptions of *GRIN2A* may account for between 9 and 20% of cases of Rolandic epilepsy^35^,^36^,^37^. Coding variants affecting *SRPX2* have also been described in patients affected by Rolandic seizures, speech dyspraxia and intellectual disability, including the same variant (p.N327S) that we found in the present study^34^. Note, however, that the discovery family with Rolandic epilepsy was subsequently found to also carry a *GRIN2A* mutation, leading some to question the role of *SRPX2* in speech apraxia^36^. The *SRPX2* p.N327S variant is also reported to exist in control individuals with a frequency of 0.26%, although these controls were not screened to exclude neurodevelopmental or speech and language difficulties^64^. *In utero* silencing of rat *Srpx2* expression has been shown to disrupt neuronal migration, as does the introduction of a mutant human protein carrying the p.N327S change^65^. Knockdown of the gene in mice has been reported to lead to reduced vocalization^66^. Clinical records did not indicate a history of seizures in our two SLI probands with variants in these genes. Our data are therefore consistent with mounting evidence that contributions of *SRPX2* to neurodevelopmental disorders are more complex than originally thought.

We also observed potential compound heterozygotes for putative disruptive variants of the *SCN9A* and *KMT2D* genes. *SCN9A* has been associated with febrile epileptic seizures, which themselves carry an increased risk of language impairment^67^. Heterozygous loss-of-function mutations of the *KMT2D* gene are implicated in Kabuki syndrome, a severe developmental syndrome that often presents with heterogeneous oromotor, speech, and language deficits^59^. The *KMT2D* variants we identified are nonsynonymous changes that may alter protein properties but are very unlikely to act as fully penetrant loss-of-function alleles, especially given that carriers of these variants do not suffer from Kabuki syndrome. Thus, if they are indeed aetiologically relevant for SLI, we must speculate that they increase risk in a subtle manner; functional assays would be required to shed more light on this hypothesis. Overall, our findings are in line with the proposed existence of shared molecular mechanisms between different neurodevelopmental disorders affecting speech and language circuits of the brain^24^.

The heterogeneity of speech and language disorders and the complexity of the underlying genetic mechanisms are further illustrated by the observation that most of our cases did not carry obvious disruptive coding variants in known genes implicated by prior literature and by the fact that few of the identified genes fell within known regions of linkage for SLI or dyslexia. Indeed, of the genes identified as candidates in this manuscript, only the *MUC6* gene falls in a previously demonstrated linkage locus (DYX7)^13^. Furthermore, although we did observe novel and rare variants in candidate language-related genes in some probands, many did not co-segregate with disorder within the family unit and their aetiological role could not be clarified, indicating that they are unlikely to be directly causal, but could perhaps increase risk of SLI in a more complex manner. Even in cases where co-segregation was established, the small size of the family units and the limitations of phenotyping in adults limit the conclusions that can be drawn. In line with current guidelines^52^, all variants would therefore require functional studies to robustly validate their relevance to SLI risk. In addition, future surveys in much larger SLI cohorts could also be informative on contributions of the various known genes to risk.

Beyond known candidate genes from the literature, we searched for variants with likely deleterious effects from elsewhere in the exome. We identified and validated two rare stop-gain variants that occurred in multiple affected children within family units. A stop-gain near the start of the *OXR1* gene was found in three siblings with speech and language-related difficulties. The OXR1 protein plays a critical role in neuronal survival during oxidative stress and is a candidate gene for amyotrophic lateral sclerosis^68^. Knockout of the *Oxr1* gene in mice leads to progressive neurodegeneration and motor-coordination deficits^69^. This gene therefore represents an interesting future candidate for involvement in neurodevelopmental disorder. A stop-gain in the *MUC6* gene was found in four siblings with expressive and receptive difficulties in another family. An important note of caution should be made here, since *MUC* genes are known to be particularly susceptible to false positive findings in next-generation sequencing studies, due to mapping artefacts (see http://massgenomics.org/2013/06/ngs-false-positives.html). As with all the other variants of interest that we discuss here, independent validation came from Sanger sequencing, still considered the gold standard method, which can increase confidence that these are not artefactual findings.

It has previously been postulated that some forms of neurodevelopmental disorder may follow a “double-hit” model in which combinations of events with relatively large effect sizes disrupt inter-connected pathways and substantially increase the risk of neurodevelopmental disorder^70^,^71^. To begin exploring this proposal with respect to SLI, we searched for genes which carried multiple rare variants of likely deleterious effect within the same proband, and probands who carried multiple events of potential interest across candidate genes. We identified several cases with multiple rare coding variants at different loci, although these did not occur in genes with obvious functional connections and they would thus need validation with further experimental data. One proband with multiple variants of interest carried a rare variant in the *AUTS2* gene in combination with a rare inherited haplotype in the *STARD9* gene. *AUTS2* is a long-standing candidate for autism susceptibility^72^ and disruptions of this gene have been reported in individuals with developmental delay^73^,^74^,^75^,^76^, ADHD^77^, epilepsy^78^ and schizophrenia^79^. Indeed, it has been described as a locus that confers risk across neurodevelopmental diagnostic boundaries^46^,^80^. The functions of the AUTS2 protein are largely unknown but it has been suggested to play a role in cytoskeletal regulation^81^. The *STARD9* gene encodes a mitotic kinesin which functions in spindle pole assembly^82^. Interestingly, another proband also carried multiple rare variants in the *STARD9* gene (Fig. 3). In both cases, the *STARD9* variants were not compound heterozygotes but instead appeared to represent inherited overlapping rare haplotypes that harboured multiple coding variants. The finding of co-occurring variants in two SLI probands leads us to speculate that pathways related to cytoskeletal function might be relevant for language disorders.

Potential involvement of cytoskeletal regulation in mechanisms underlying SLI susceptibility was also suggested by our independent pathway-based investigations of the exome datasets. GO analyses between and within probands converged on biological processes including microtubule-based movement, specifically the roles of dyneins and kinesins. These findings thus suggest an intriguing link between the specific variants identified in single probands and the patterns of variants seen across all probands. In addition, certain biological functions appeared to cluster within variant frequency groupings. While novel and rare (0–1%) variants were over-represented within “Extracellular matrix disassembly” pathways, more common variants (1–5%) were predominantly localized within the “Microtubule-based movement” class. A potential contribution of microtubule transport pathways to risk of speech and language problems would be of particular interest given the established links between candidate genes for neurodevelopmental disorders and dynein and cilia function^20^,^65^,^83^,^84^,^85^,^86^.

The GO categories identified as being over-represented are large functional classes and the sample sizes are small, but these analyses provide preliminary indications of pathways that may be relevant to speech and language disorders. Further investigations of larger samples will be required to validate these initial findings and to elucidate whether particular subsets of genes are enriched with risk variants or whether the risk is distributed across the entire class.

The ultimate aim of exome studies is to perform an unbiased screen of all variants across the entire coding sequence. Given the sample size of the present study, we used a number of complementary methods to constrain searches for variants of interest and associated pathways. It is therefore important to note that our analyses necessarily highlight a constricted subset of loci that have supporting data from previous datasets or have an increased likelihood of aetiological significance. We have listed all identified variants within each category in the Tables presented here and as Supplementary data. Nonetheless, these analyses have enabled the detection of cases with potentially pathogenic mutations (*ERC1, GRIN2A, SRPX2*), and support the role of known candidate genes and pathways (*AUTS2*, ciliary function). Moreover, our findings highlight a number of new putative candidates for future study (e.g. *OXR1, STARD9*) and novel pathways and processes (microtubule transport, cytoskeletal regulation) that may be relevant to speech and language development.

## Methods

### Participants

Participants for this study were taken from the SLIC (SLI consortium) cohort, the ascertainment and phenotyping of which has been described extensively in prior publications^7^,^17^,^51^,^60^,^87^,^88^ and were recruited from five centres across the UK; The Newcomen Centre at Guy’s Hospital, London (now called Evelina Children’s Hospital); the Cambridge Language and Speech Project (CLASP); the Child Life and Health Department at the University of Edinburgh; the Department of Child Health at the University of Aberdeen; and the Manchester Language Study. A full list of SLIC members can be found in the Acknowledgements section. All methods were performed in accordance with the relevant ethical guidelines and regulations. Ethical agreement was given by local ethics committees. Guys Hospital Research Ethics Committee approved the collection of families from the Newcomen Centre to identify families from the South East of England with specific language disorder, Ref. No. 96/7/11. Cambridge Local Research Ethics Committee approved the CLASP project “Genome Search for susceptibility loci to language disorders”, Ref. No. LREC96/212. Ethical approval for the Manchester Language Study was given by the University of Manchester Committee on the Ethics of Research on Human Beings, Ref. No. 03061. The Lothian Research Ethics Committee approved the project “Genetics of specific language impairment in children in Scotland”, Ref. No. LREC/1999/6/20. All subjects provided informed consent.

Briefly, the SLIC cohort comprises a set of British nuclear families who were recruited through at least one child with a formal diagnosis of SLI. This diagnosis was based on impaired expressive and/or receptive language skills (≥1.5 standard deviations (SD) below the normative mean of the general population), assessed using the Clinical Evaluation of Language Fundamentals (CELF-R)^89^. The language impairments had to occur against a background of normal non-verbal cognition (not more than 1 SD below that expected for their age), assessed using the Perceptual Organisation Index (a composite score derived from Picture Completion, Picture Arrangement, Block Design and Object Assembly subtests) of the Wechsler Intelligence Scale for Children (WISC)^90^. Following recruitment of the proband, language and IQ measures were collected for all available siblings, regardless of language ability and DNA samples were collected from parents and children. Crucially, although there have been reports of linkage^7^,^87^,^88^, association^17^,^31^,^51^,^61^,^91^ and CNV analyses^60^,^92^,^93^ of the SLIC families, no prior investigation has used exome-wide next-generation sequencing approaches to investigate etiology in this cohort. For the present study, we first selected unrelated probands from the SLIC cohort who had severe SLI, based on in-depth phenotypic data on multiple measures of language and cognition, along with sufficient quantities of high-quality DNA available for next-generation sequencing. This yielded a set of forty three unrelated probands for whom whole exome sequencing was carried out. The group of probands had mean scores of 65.9 (−2.3 SD below expected for chronological age) and 73.8 (−1.7 SD) for expressive and receptive language respectively, and a mean verbal IQ of 84.2 (−1.1 SD), compared to a mean non-verbal IQ of 98.7 (−0.1 SD) in line with the mean of the general population (all scores normalized to a population mean of 100 and SD of 15).

In our Figures examining family segregation of variants (see below) we present information regarding the core phenotypes; CELF-R expressive and receptive language scores, which were used to determine proband and sibling affection status. Where available, we also present data for additional phenotypes. These include the total verbal and non-verbal IQ scores from the Wechsler Intelligence Scale for Children^90^ and scores on nonword repetition tasks^94^. Although these were not used to ascertain affection status, they sometimes provided additional information regarding specific deficits in individuals. Nonword repetition is hypothesized to represent an index of phonological short term memory, while the IQ measures indicate general levels of verbal and non-verbal ability.

### Exome sequencing and variant discovery

Exome capture was performed using 10 μg of genomic DNA from each participant. Exons and flanking intronic regions were captured with the SureSelect Human All Exon version-2 50 Mb kit (Agilent, Santa Clara, CA, USA), which is designed to capture 99% of human exons defined by NCBI Consensus CDS Database from September 2009, and 93% of RefSeq genes (~23,000). Captured fragments were sequenced using the SOLiD series 5500xl DNA sequencing platform (Life Technologies, Carlsbad, CA, USA) with 50 nt, single-end runs. Sequence alignment and variant calling were performed within the GenomeAnalysis Toolkit (GATK version-2.7.2)^95^. BAM files went through several stages of preprocessing, including removal of PCR duplicates using Picard Tools version-1.77 (URL: http://picard.sourceforge.net/), Base Quality Recalibration, and Indel Realignment (which form part of the GATK software package). Calling of single nucleotide variants (SNVs) was performed using a combined calling algorithm with HaplotypeCaller, which can provide a better stringency of calling and more accurate estimation of variant quality.

Raw variant calls were filtered using the Variant Quality Score Recalibration function according to GATK’s Best Practice recommendations^50^, with the following training sets: human hapmap-3.3.hg19 sites, 1000G-omni-2.5.hg19 sites, and 1000G-phase1-high.confidence-SNPs.hg19 sites for SNVs, and Mills-and-1000G-gold.standard-INDELs.hg19 for INDELs. Using this training set, variant call files are recalibrated and filtered according to various parameters including the normalization of read depth (QD), the position of the variant within the read (ReadPosRankSum), the mapping quality of variant call reads (MQRankSum), strand bias (FS), and inbreeding coefficients (InbreedingCoeff). The PASS threshold after recalibration was set at 99 (99% of the testing dbSNP-137 variants could be identified using the trained model).

Filtered variants were annotated according to coordinates of human genome build hg19, RefSeq genes and dbSNP137 using the ANNOVAR annotation tool^96^ which enables gene-based (e.g. functional consequence of identified changes), region-based (e.g. segmental duplications, DNAse hypersensitive sites) and filter-based (e.g. population frequencies, SIFT scores) annotations. Following annotation, all intergenic, intronic, non-coding RNAs, synonymous variants, changes that fell within a region of known segmental duplication and variants with sequencing depth below 10 in all probands were excluded from further analysis. The numbers of variants remaining at each filtering stage are shown in Supplementary Table S2. Allele frequencies were derived from 1000 Genomes Phase I (v2) data (Apr 2012) (ftp://ftp.1000genomes.ebi.ac.uk/vol1/ftp/technical/working/20120316_phase1_integrated_release_version2/) and the Exome Variants Server (evs.gs.washington.edu/esp5500_bulk_data/ESP5400.snps.vcf.tar.gz) for all analyses described throughout the paper. These databases include sequence data for >5500 individuals allowing us to detect variants with expected allele frequencies >0.009%.

The primary data for the study are deposited at The Language Archive (TLA: https://corpus1.mpi.nl/ds/asv/?0), a public data archive hosted by the Max Planck Institute for Psycholinguistics. Data are stored at the TLA under the node ID: MPI2010433#, and accessible with a persistent identifier: https://hdl.handle.net/1839/00-0000-0000-001E-AD41-2@view. Access can be granted upon request. TLA content is also visible from the Data Archiving and Networked Services (DANS) database, the Dutch national organization for sustained access to digital research data.

### Variants of potential aetiological significance: selection, validation and segregation

Beyond two very recent studies targeting geographically isolated populations^48^,^49^, extensive investigations of exome data in individuals affected by SLI have not previously been completed. The first stage of our analyses involved the identification of sets of variants of potential aetiological significance. In accordance with current guidelines^52^, we employed several complementary approaches, which considered public data sets and previously published data and employed multiple metrics followed by targeted validation and cosegregation analyses, as detailed below:

- We considered all coding variants identified within a set of the most robust candidate genes from the literature, defined prior to the start of the analysis. This set included 19 genes (*CMIP, ATP2C2, CNTNAP2, NFXL1, FOXP1, FOXP2, DYX1C1, KIAA0319, DCDC2, ROBO1, SRPX2, GRIN2A, GRIN2B, ERC1, SETBP1, CNTNAP5, DOCK4, SEMA6D*, and *AUTS2*), as detailed in the main text.
- We identified rare variants (frequency of ≤1% in 1000 Genomes) that conferred stop-gain mutations that were predicted to be deleterious (SIFT score ≤0.05 or PolyPhen2 score ≥0.85) and that passed all the filters listed in Supplementary Table S2.
- We searched for potential compound heterozygotes by identifying all probands who carried two or more rare coding variants in a single gene. These variants were filtered to include only nonysynonymous or stop-gain/loss variants, splice-site changes and frame-shift INDELs that were novel or rare (frequency of ≤1% in 1000 Genomes (ALL)^97^,^98^ and the NHLBI GO ESP Exome Variant Server (EVS, ESP5400, ALL samples) http://evs.gs.washington.edu/EVS/)), and that were predicted to be deleterious (SIFT score ≤0.05 or PolyPhen2 score ≥0.85). Variants that fell in regions of segmental duplication or within 10 bp of each other were excluded. Segregation analyses (see below) then enabled us to decipher whether the rare coding variants in the proband occurred on the same, or a different, chromosomal copy, to determine which cases were most likely to be compound heterozygotes.
- We highlighted potential cases of “multiple-hits” by following up all probands who had more than one variant which fell into any of the above classes of investigation.

All the above variants were validated by Sanger sequencing within the probands in whom they were called. Validated variants of interest were then also sequenced in all available parents and siblings of the proband allowing the evaluation of possible segregation patterns within nuclear pedigrees.

### Pathway-based analyses

In the second stage of analyses, we performed a more exploratory investigation of biological pathways within the exome dataset. For each proband, we collated a list of all genes containing rare likely disruptive variants, defined as nonysynonymous and stop-gain/loss variants, splice-site changes and frame-shift INDELs that had a frequency of ≤1% in 1000 Genomes (ALL)^97^,^98^ and the NHLBI GO ESP Exome Variant Server (EVS, ESP5400, ALL samples) http://evs.gs.washington.edu/EVS/)) and that were predicted to be deleterious (SIFT score ≤ 0.05 or PolyPhen2 score ≥ 0.85) (2,818 variants in total for all probands, Supplementary Table S6). We then used the KEGG^99^ and Reactome^100^ databases to identify pathways affected by these variants within probands. To test whether the observed number of SLI probands sharing a particular affected pathway was higher than chance, random subject-gene associations were generated, by picking the same number of genes randomly from all genes with variants. Thus, a permuted pathway-to-subjects mapping was generated by repeating the process 1000 times. The FDR was calculated as the number of times when a pathway was seen in equal or more probands than the observed probands divided by 1000.

Following this within-proband analyses, we went on to perform gene ontology (GO) analyses in the dataset as a whole. A list of all genes containing rare and disruptive variants (defined as above, based on 2,818 variants, Supplementary Table S6) was tested against the background gene list (all genes with all variants). Over-represented classes were identified across all probands using the GO database^101^ and hypergeometric tests were conducted within GOstats^102^ using a P-value- and FDR-level of 0.01.

Finally, we examined effects of variant frequency upon gene pathways. For these analyses, we focused on all nonsynonymous, stop-gain and stop-loss variants that had a frequency of ≤5% in 1000 Genomes (ALL)^97^,^98^, regardless of functional predictions. From this list we selected genes which carried novel variants i.e. variants that were not found in 1000 Genomes and not found in EVS (a total of 3,876 variants, as listed in Supplementary Table S7). The remaining genes were split into (i) genes that carried variants that had been reported in the 1000 Genomes with a variant frequency of <1% (7,084 variants, Supplementary Table S8) and, (ii) genes which carried variants with an 1000 Genomes frequency of between 1% and 5% (4,971 variants, Supplementary Table S9).

## Additional Information

**How to cite this article:** Chen, X. S. *et al*. Next-generation DNA sequencing identifies novel gene variants and pathways involved in specific language impairment. *Sci. Rep.* **7**, 46105; doi: 10.1038/srep46105 (2017).

**Publisher's note:** Springer Nature remains neutral with regard to jurisdictional claims in published maps and institutional affiliations.

## Supplementary Material

Supplementary Figures

Supplementary Tables

## Figures and Tables

**Table 1:** Number of validated calls in candidate genes in SLIC probands.

Gene	Validated calls with pop freq >5%^a^	Validated calls with pop freq 1–5%^a^	Validated calls with pop freq <1%^a^	Novel validated calls^b^	Total validated calls
*ATP2C2*	2	2	2	0	6
*AUTS2*	1	0	1	0	2
*CMIP*	0	0	0	0	0
*CNTNAP2*	0	0	0	1	1
*CNTNAP5*	0	1	1	0	2
*DCDC2*	2	1	0	0	3
*DOCK4*	0	0	0	0	0
*DYX1C1*	0	0	0	0	0
*ERC1*	1	1	0	1	3
*FOXP1*	0	0	0	0	0
*FOXP2*	0	0	0	0	0
*GRIN2A*	0	0	0	1	1
*GRIN2B*	0	0	0	1	1
*KIAA0319*	4	2	0	0	6
*NFXL1*	1	0	0	0	1
*ROBO1*	0	1	1	0	2
*SEMA6D*	2	0	0	1	3
*SETBP1*	5	0	0	0	5
*SRPX2*	0	0	1	0	1
All	18 (48.6%)	8 (21.6%)	6 (16.2%)	5 (13.5%)	37

^a^Population frequency is taken from 1000 Genomes (Apr2012_ALL) samples.

^b^Novel variants were not described by 1000 Genomes (Apr2012_ALL) or by the exome variant server (ESP5400_ALL). A full list of all 37 variants can be found in Supplementary Table S4.

**Table 2:** Novel and rare variants in candidate genes in SLIC probands.

Variant Position	Gene	dbSNP137	Ref	Var	Proband IDs	EVS (5400_ALL) variant freq	1000G (ALL Apr2012) variant freq	Variant status	coding change	PhyloP	Phast Cons	SIFT	Poly Phen	Comments
chr2:125504881	*CNTNAP5*	rs35085748	T	C	39	0.78%	0.64%	Rare	NM_130773:exon14: c.T2150C:p.V717A	1.27	0.93	0.59	0.00	
chr3:78766524	*ROBO1*	rs80030397	A	G	17	0.02%	0.05%	Rare	NM_001145845:exon5: c.T701C:p.V234A	4.77	1.00	0.17	1.00	
chr7:69364311^a^	*AUTS2*	rs142957106	C	T	19	0.08%	NA	Rare	NM_001127231:exon2: c.C349T:p.R117C	2.84	1.00	0.02	1.00	
chr7:146829358	*CNTNAP2*	rs368057493^b^	G	T	40	NA	NA	Novel	NM_014141:exon8: c.G1105T:p.V369L	5.44	1.00	0.35	0.00	
**chr12**:**1137072**	***ERC1***		**G**	**A**	**23**	**NA**	**NA**	**Novel**	**NM_178039**:**exon2**: **c**.**G3A**:**p**.**M1I**	**6**.**15**	**1**.**00**	**0**.**00**	**0**.**91**	**START**-**LOSS**
chr12:13715865	*GRIN2B*		C	G	25	NA	NA	Novel	NM_000834:exon13: c.G4307C:p.G1436A	1.29	1.00	0.90	0.00	
chr15:48063365	*SEMA6D*^*a*^		C	G	30	NA	NA	Novel	NM_020858:exon17: c.C2419G:p.H807D	5.70	1.00	0.26	0.49	
**chr16**:**9916226**	***GRIN2A***		**C**	**G**	**4**	**NA**	**NA**	**Novel**	**NM_001134407**:**exon10**: **c**.**G2063C**:**p**.**G688A**	**5**.**94**	**1**.**00**	**0**.**00**	**1**.**00**	***DE NOVO***
chr16:84438827	*ATP2C2*	rs78887288	G	A	35	0.46%	0.14%	Rare	NM_001286527:exon3: c.G304A:p.V102M	0.24	0.09	0.11	0.03	
chr16:84494315	*ATP2C2*	rs62050917	C	T	27	0.79%	0.41%	Rare	NM_001291454:exon21: c.C1936T:p.R646W	−1.51	0.00	0.00	0.99	
36														
39														
**chrX**:**99922289**	***SRPX2***	**rs121918363**	**A**	**G**	**41**	**0**.**08%**	**NA**	**Rare**	**NM_014467**:**exon9**: **c**.**A980G**:**p**.**N327S**	**1**.**37**	**0**.**92**	**0**.**00**	**0**.**06**	**HGMD ID CM061219**

Scores shown in bold & italic represent changes that are predicted to be functionally significant. Variants highlighted in bold represent events of putative significance (see Fig. 1 for family pedigrees).

^a^Family pedigree shown in Fig. 3.

^b^dbSNP number exists, but no frequency information in EVS or 1000G.

**Table 3:** Stop-gain variants identified in SLIC probands.

Variant Position	Gene	dbSNP137	Ref	Var	Proband ID	1000G (ALL) variant freq	EVS (5400_ALL) variant freq	Variant status	Coding change	% of protein missing	PhyloP	Phast Cons	SIFT
chr1:158532597	*OR6P1*	rs142215019	G	T	34	0.10%	0.22%	rare	NM_001160325:exon1: c.C798A:p.Y266X	16.4%	−0.62	0.00	1.00
chr3:63466576^a^	*SYNPR*	rs376661036	C	A	30	NA	0.01%	rare	NM_144642:exon2: c.C93A:p.C31X	84.7%	−0.72	0.60	1.00
**chr8**:**107738486**	***OXR1***	**rs145739822**	**G**	**A**	**29**	**0**.**14%**	**NA**	**rare**	**NM_001198534**:**exon1**: **c**.**G15A**:**p**.**W5X**	**97**.**7%**	**4**.**24**	**1**.**00**	**1**.**00**
chr8:39847306	*IDO2*	rs199869245	C	T	11	NA	0.05%	rare	NM_194294:exon8: c.C655T:p.R219X	49.5%	1.90	0.95	1.00
**chr11**:**1027390**	***MUC6***	**rs200217410**	**G**	**T**	**8**	**NA**	**0**.**06%**	**rare**	**NM_005961**:**exon17**: **c**.**C2109A**:**p**.**C703X**	**71**.**2%**	**0**.**40**	**1**.**00**	**1**.**00**
chr11:6190828^a^	*OR52B2*	rs190537696	A	T	19	0.14%	0.10%	rare	NM_001004052:exon1: c.T729A:p.C243X	25.0%	0.59	1.00	1.00
chr16:4745030	*NUDT16L1*	rs146701095	C	T	9	0.05%	0.04%	rare	NM_001193452:exon3: c.C556T:p.Q186X	3.6%	1.77	1.00	1.00

Scores shown in bold & italic represent changes that are predicted to be functionally significant. Variants highlighted in bold represent co-segregating stop-gains (see Figs 2 and 3 for family pedigrees).

^a^Family pedigree shown in Fig. 3.

**Table 4:** Genes with more than one rare variant in the same SLIC proband.

Variant Position	Gene	dbSNP137	Ref	Var	Proband IDs	1000G (ALL) variant freq	EVS variant freq	Variant status	Coding change	PhyloP	Phast Cons	SIFT	Poly Phen	Notes
**chr2**:**167089942**	***SCN9A***	**rs180922748**	**G**	**C**	**2**	**0**.**14%**	**0**.**20%**	**rare**	**NM_002977**:**exon21**:**c**.**C3799G**:**p**.**L1267V**	**2**.**07**	**1**.**00**	**0**.**13**	**0**.**99**	
**chr2**:**167094638**	***SCN9A***	**rs141268327**	**T**	**C**		**0**.**41%**	**0**.**53%**	**rare**	**NM_002977**:**exon20**:**c**.**A3734G**:**p**.**N1245S**	**4**.**93**	**1**.**00**	**0**.**00**	**1**.**00**	
chr2:32689842	*BIRC6*	rs61754195	C	T	26, 42	0.46%	0.98%	rare	NM_016252:exon25:c.C5207T:p.P1736L	3.09	0.99	0.01	0.60	
chr2:32740353	*BIRC6*	rs61757638	C	T		0.18%	0.25%	rare	NM_016252:exon55:c.C10865T:p.A3622V	6.06	1.00	0.00	0.99	
chr3:58104626	*FLNB*	rs139875974	G	T	40	0.05%	0.07%	rare	NM_001164317:exon19:c.G2773T:p.G925C	6.33	1.00	0.00	1.00	
chr3:58110119	*FLNB*	rs111330368	G	C		0.23%	0.41%	rare	NM_001164317:exon22:c.G3785C:p.G1262A	6.18	1.00	0.00	1.00	
chr11:6190710	*OR52B2*^*a*^	rs372373798	C	T	19	NA	0.01%	novel	NM_001004052:exon1:c.G847A:p.V283M	2.69	1.00	0.08	1.00	
chr11:6190828	*OR52B2*^*ab*^	rs190537696	A	T		0.14%	0.10%	rare	NM_001004052:exon1:c.T729A:p.C243X	0.59	1.00	1.00	.	STOP
**chr11**:**92086828**	***FAT3***	**rs139595720**	**T**	**C**	**7**	**0**.**46%**	**0**.**66%**	**rare**	**NM_001008781**:**exon1**:**c**.**T1550C**:**p**.**L517S**	**3**.**32**	**0**.**82**	**0**.**72**	**1**.**00**	
**chr11**:**92624235**	***FAT3***	**rs187159256**	**C**	**T**		**0**.**14%**	**0**.**17%**	**rare**	**NM_001008781**:**exon25**:**c**.**C13630T**:**p**.**L4544F**	**0**.**94**	**0**.**96**	**0**.**03**	**0**.**37**	
**chr12**:**49418717**	***KMT2D***^***a***^	**rs201481646**	**C**	**T**	**12**	**NA**	**0**.**07%**	**rare**	**NM_003482**:**exon49**:**c**.**G15797A**:**p**.**R5266H**	**2**.**39**	**1**.**00**	**0**.**00**	**1**.**00**	
**chr12**:**49432365**	***KMT2D***^***a***^	**rs199547661**	**G**	**A**		**0**.**09%**	**0**.**24%**	**rare**	**NM_003482**:**exon34**:**c**.**C8774T**:**p**.**A2925V**	**0**.**77**	**0**.**38**	**0**.**00**	**0**.**00**	
chr13:109613971	*MYO16*	rs374252281	G	A	28	NA	0.01%	rare	NM_001198950:exon18:c.G2122A:p.A708T	4.62	1.00	0.01	1.00	
chr13:109617108	*MYO16*		G	A		NA	NA	novel	NM_001198950:exon20:splice acceptor lost	4.87	1.00	.	.	SPLICE
chr14:58924684	*KIAA0586*^*a*^	rs61742715	T	A	19	0.23%	0.39%	rare	NM_001244189:exon13:c.T1729A:p.L577I	0.28	0.81	0.43	1.00	
chr14:59014632	*KIAA0586*^*a*^	rs61745066	G	A		0.18%	0.24%	rare	NM_001244189:exon34:c.G4873A:p.G1625R	−1.15	0.41	0.00	0.00	
chr15:42977116	*STARD9*^*a*^	rs79165890	T	C	12	0.05%	0.22%	rare	NM_020759:exon23:c.T3340C:p.C1114R	1.98	0.51	0.00	0.05	
chr15:42977810	*STARD9*^*a*^	rs140924205	T	G	19	0.32%	0.40%	rare	NM_020759:exon23:c.T4034G:p.I1345S	0.269	0.001	0.00	0.27	
chr15:42978141	*STARD9*^*a*^	rs376229251	A	C	19	NA	0.09%	rare	NM_020759:exon23:c.A4365C:p.E1455D	0.553	0.067	0.00	0.99	
chr15:42981101	*STARD9*^*a*^	rs202017657	C	G	12, 19	NA	0.15%	rare	NM_020759:exon23:c.C7325G:p.P2442R	0.28	0.01	0.00	0.99	
chr15:42982237	*STARD9*^*a*^	rs201340789	G	C	12, 19	NA	0.19%	rare	NM_020759:exon23:c.G8461C:p.V2821L	0.28	0.01	0.00	0.99	
**chr16**:**23635348**	***PALB2***	**rs45478192**	**A**	**C**	**13**	**0**.**09%**	**0**.**17%**	**rare**	**NM_024675**:**exon8**:**c**.**T2816G**:**p**.**L939W**	**2**.**83**	**1**.**00**	**0**.**00**	**1**.**00**	
**chr16**:**23641275**	***PALB2***	**rs45543843**	**T**	**A**	**NA**	**0**.**01%**	**rare**	**NM_024675**:**exon5**:**c**.**A2200T**:**p**.**T734S**	**2**.**90**	**0**.**97**	**0**.**11**	**1**.**00**		
chr17:34861135	*MYO19*	rs200572125	C	T	25	NA	0.03%	rare	NM_001163735:splice donor lost, exon20	4.98	1.00	.	.	SPLICE
chr17:34871802	*MYO19*	rs187710120	T	C		0.05%	0.19%	rare	NM_001163735:exon8:c.A446G:p.Y149C	4.52	1.00	0.00	1.00	

Scores shown in bold & italic represent changes that are predicted to be functionally significant. Variants highlighted in bold represent potential compound heterozygotes.

^a^Family pedigree shown in Fig. 3.

^b^Stop-gain, also represented in Table 3.

**Table 5:** Enriched GO terms with variants less than 1% frequency across probands.

Term	ExpCount	Count	P-value	FDR
**ciliary or bacterial**-**type flagellar motility**	3.63	12	8.33E-05	0.012
**microtubule**-**based movement**	20.46	37	0.000197	0.009
cell adhesion	125.70	160	0.000543	0.005
homophilic cell adhesion	18.71	33	0.000683	0.008
actin cytoskeleton organization	55.05	78	0.000777	0.004
**extracellular matrix organization**	46.57	65	0.002975	0.011
protein depolymerization	8.75	17	0.004587	0.001
cellular component assembly involved in morphogenesis	20.99	33	0.005049	0.003
dendrite development	17.23	28	0.005803	0.002
double-strand break repair via homologous recombination	6.99	14	0.007348	0.004
**neuromuscular junction development**	4.98	11	0.007624	0.005
actin polymerization or depolymerization	15.48	25	0.00954	0.006
cell projection organization	126.51	151	0.009759	0.000

Pathways with P < 0.01 and size >10 genes are shown in the table. Those in bold are also found to be significantly enriched when considering a relaxed gene list over different variant frequencies, shown in Table 6.

**Table 6:** Enriched GO terms across probands split by variant frequency.

Term	ExpCount	Count	Pvalue	FDR
**Novel**				
cellular response to interleukin-4	5.42	13	2.35E-04	0.006
maintenance of location in cell	24.07	40	2.41E-04	0.004
keratinization	3.85	10	9.66E-04	0.004
microtubule anchoring	7.94	15	0.0052	0.012
neuromuscular junction development	8.96	16	0.0078	0.003
release of sequestered calcium ion into cytosol by sarcoplasmic reticulum	4.09	9	0.0090	0.002
**Frequency between 0 and 1%**				
extracellular matrix disassembly	23.67	41	8.57E-05	0.004
cell proliferation in forebrain	3.79	11	1.67E-04	0.004
mitotic sister chromatid segregation	11.36	23	2.06E-04	0.006
cilium assembly	20.12	34	2.16E–04	0.01
microtubule anchoring	7.34	16	6.78E-04	0.005
cerebral cortex cell migration	4.26	11	7.53E-04	0.003
neuromuscular junction development	8.29	16	0.0034	0.008
ciliary or bacterial-type flagellar motility	6.15	13	0.0031	0.004
**Frequency between 1% and 5%**				
microtubule-based movement	46.38	76	1.81E-07	0.004
homophilic cell adhesion	45.69	74	3.26E-07	0.006
ciliary or bacterial-type flagellar motility	9.00	19	7.06E-05	0.005
release of sequestered calcium ion into cytosol	17.65	31	1.16E-04	0.006
regulation of sequestering of calcium ion	17.65	31	1.16E-04	0.006
chemokine-mediated signaling pathway	9.35	18	6.74E-04	0.004
regulation of release of sequestered calcium ion into cytosol by sarcoplasmic reticulum	4.73	10	0.0055	0.005
sarcoplasmic reticulum calcium ion transport	6.23	12	0.0053	0.003

Pathways with P < 0.01 and size >10 genes are shown in the table. Highlighted key words indicate functions consistently found in all GO enrichment analysis.
