# Supplementary material for: Next-generation DNA sequencing identifies novel gene variants and pathways involved in specific language impairment
Source: Sci Rep. 2017 Apr 25;7:46105. doi: 10.1038/srep46105 (PMC5404330; doi:10.1038/srep46105)
Supplement: Supplementary Figures [file srep46105-s1.pdf]

<u>Next-generation sequencing identifies novel gene variants and pathways involved in specific language impairment</u>

Xiaowei Sylvia Chen, Rose H. Reader, Alexander Hoischen, Joris A. Veltman, Nuala H. Simpson, Clyde Francks, Dianne F. Newbury, Simon E. Fisher

<u>SUPPLEMENTARY FIGURES</u>

SUPPLEMENTARY FIGURE S1 - Coverage versus read depth of 43 SLI samples
SUPPLEMENTARY FIGURE S2 - Rare and Novel Variants in Candidate Genes
SUPPLEMENTARY FIGURE S3 - STOP-gain variants identified in SLIC probands
SUPPLEMENTARY FIGURE S4 - Probands with multiple novel or rare variants in one gene

<u>SUPPLEMENTARY TABLES – please see excel file</u>

SUPPLEMENTARY TABLE S1 - Sequence metrics for 43 SLI samples
SUPPLEMENTARY TABLE S2 - Number of variants at each filtering stage
SUPPLEMENTARY TABLE S3 - Numbers of rare and novel variants per individual
SUPPLEMENTARY TABLE S4 - Coding and splice-site variants in candidate genes
SUPPLEMENTARY TABLE S5 - Protein interaction pathways shared by multiple SLI probands
SUPPLEMENTARY TABLE S6 - Variants included in GO analyses of transcripts carrying high priority rare or novel coding SNVs between and across probands
SUPPLEMENTARY TABLE S7 - Variants included in GO analyses of transcripts carrying novel coding SNVs across probands
SUPPLEMENTARY TABLE S8 - Variants included in GO analyses of transcripts carrying rare (<1%) coding SNVs across probands
SUPPLEMENTARY TABLE S9 - Variants included in GO analyses of transcripts carrying coding SNVs with expected frequencies between 1% and 5% across probands

SUPPLEMENTARY FIGURE S1 - Coverage versus read depth of 43 SLI samples

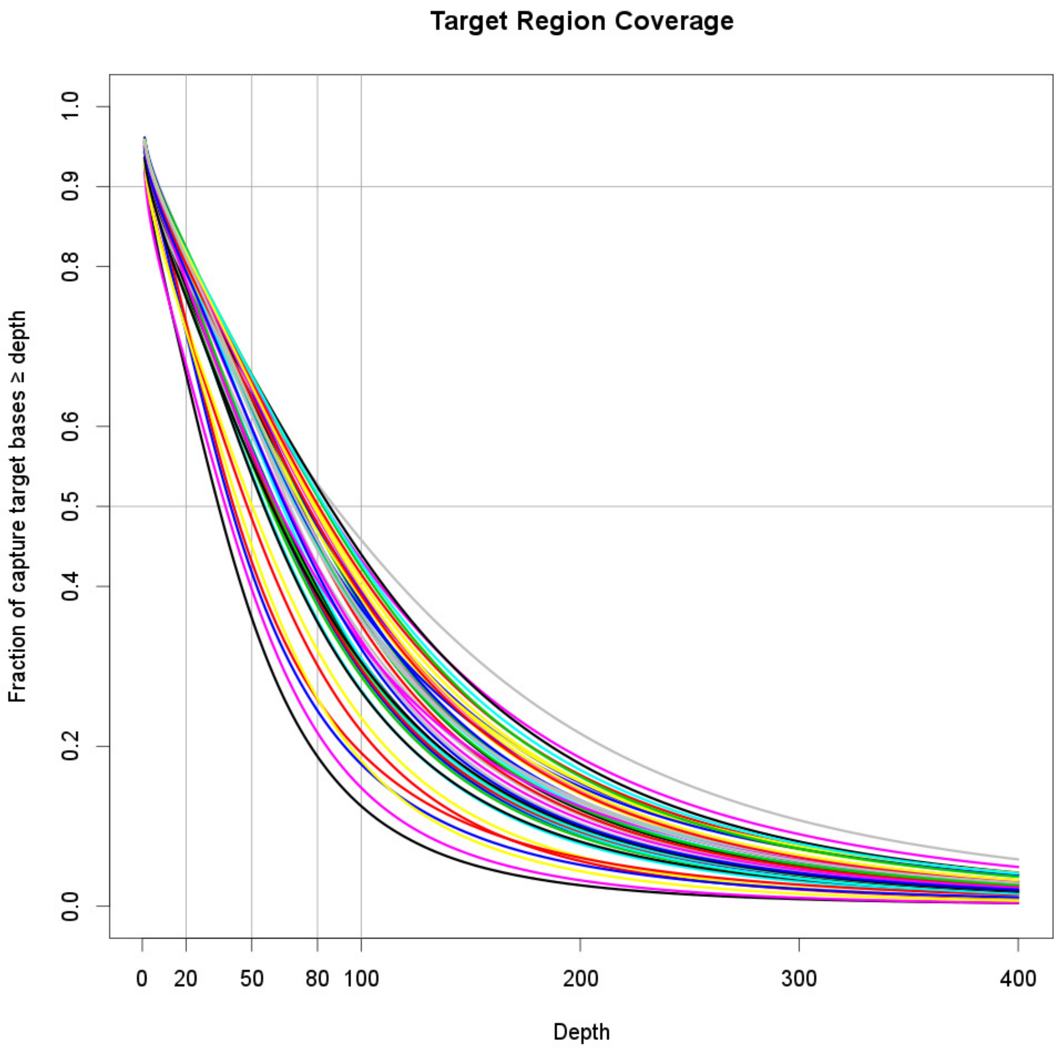

<u>SUPPLEMENTARY FIGURE S2 - Rare and Novel Variants in Candidate Genes</u>
Variants are shown in map order with '+' denoting variant carrier. Variants of putative significance are shown in Figure 1 of the main manuscript. Variants that co-occur with other variants of putative significance are shown in Figure 3 of the main manuscript.

chr2: 125504881, Proband 39 – *CNTNAP5*, V717A
Variant was not genotyped in family (EVS_EA>1%)

chr3:78766524, Proband 17 – *ROBO1*, V234A

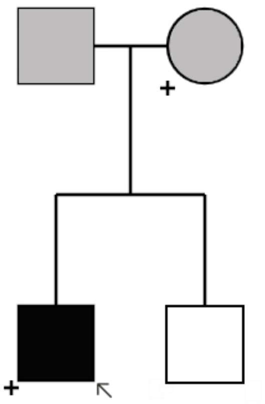

	Father	Mother	Proband	Sib
**Gtyp**	AA	AG	AG	AA
**ELS**			**50**	80
**RLS**			80	103
**NWR**	55	84	87	**58**
**VIQ**			NA	NA
**PIQ**			110	89

chr7:69364311, Proband 19 – *AUTS2*, R117C
Pedigree shown in Figure 3

chr7:146829358, Proband 40 – *CNTNAP2*, V369L

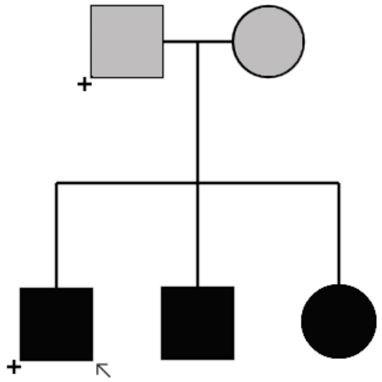

	Father	Mother	Proband	Sib1	Sib2
**Gtyp**	GT	GG	GT	GG	GG
**ELS**			**72**	80	**65**
**RLS**			99	**74**	69
**NWR**	88	78	85	89	92
**VIQ**			99	101	93
**PIQ**			104	121	**67**
Both parents self-report a history of speech and language difficulties					

chr12: 1137072, Proband 23 – *ERC1*, M1I
Pedigree shown in Figure 1

SUPPLEMENTARY FIGURE S2 (cont.) - Rare and Novel Variants in Candidate Genes

chr12:13715865, Proband 25 – *GRIN2B*, G1436A

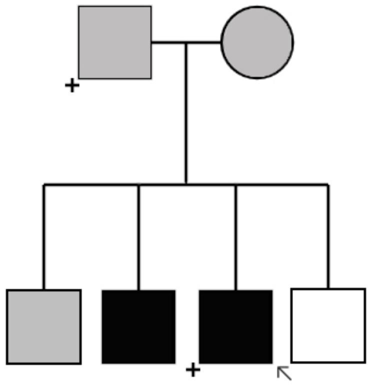

	Father	Mother	Sib1	Sib2	Proband	Sib3
**Gtyp**	CG	CC	CC	CC	CG	CC
**ELS**				**76**	**64**	100
**RLS**				95	**67**	104
**NWR**				104	96	104
**VIQ**				91	80	86
**PIQ**				101	109	112

Father self-reports a history of speech and language difficulties

chr15:48063365, Proband 30 – *SEMA6D*, H807D
Pedigree shown in Figure 3

chr16:9916226, Proband 4 – *GRIN2A*, G688A
Pedigree shown in Figure 1

chr16:84438827, Proband 35 – *ATP2C2*, V102M

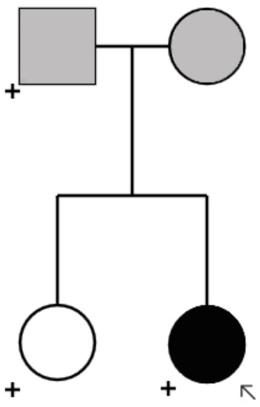

	Father	Mother	Sib	Proband
**Gtyp**	GA	GG	GA	GA
**ELS**			91	**50**
**RLS**			112	**50**
**NWR**			90	
**VIQ**			100	**63**
**PIQ**			103	82

chr16:84494315, Probands 27, 36 & 39 – *ATP2C2*, R646W
Variant was not genotyped in families (EVS_EA>1%)

chrX:99922289, Proband 41 – *SRPX2*, N327S
Pedigree shown in Figure 1

SUPPLEMENTARY FIGURE S3 - STOP-gain variants identified in SLIC probands

Variants are shown in map order with '+' denoting variant carrier. Stop-gains that co-segregate with disorder are shown in Figure 2 of the main manuscript. Variants that co-occur with other variants of putative significance are shown in Figure 3 of the main manuscript.

chr1:158532597, Proband 34 – *OR6P1*, Y266X

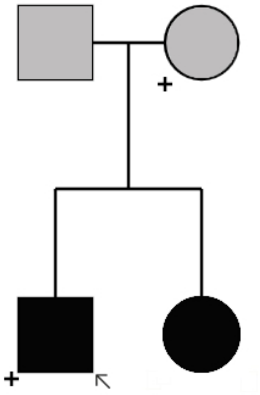

	Father	Mother	Proband	Sib
**Gtyp**	GG	GT	GT	GG
**ELS**			**50**	**50**
**RLS**			**54**	**50**
**NWR**	84	88	102	**62**
**VIQ**			83	**69**
**PIQ**			80	99

chr3:63466576, Proband 30 – *SYNPR*, C31X

Pedigree shown in Figure 3

chr8:39847306, Proband 11 – *IDO2*, R219X

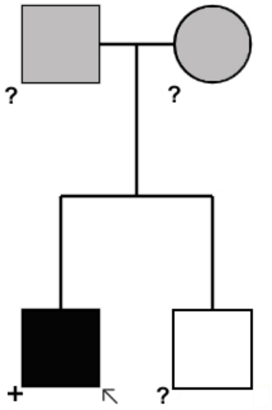

	Father	Mother	Proband	Sib
**Gtyp**	NA	NA	CT	NA
**ELS**			**72**	
**RLS**			**76**	
**NWR**			**75**	
**VIQ**				
**PIQ**			89	89

chr8:107738486, Proband 29 – *OXR1*, W5X

Pedigree shown in Figure 2

SUPPLEMENTARY FIGURE S3 (cont.) - STOP-gain variants identified in SLIC probands

chr11:1027390, Proband 8 – *MUC6*, C703X
Pedigree shown in Figure 2

chr11:6190828, Proband 19 – *OR52B2*, C243X
Pedigree shown in Figure 3

chr16:4745030, Proband 9 – *NUDT16L1*, Q186X

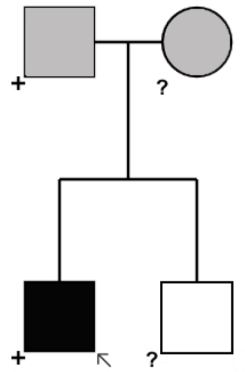

	Father	Mother	Proband	Sib
**Gtyp**	CT	NA	CT	NA
**ELS**			**54**	78
**RLS**			**59**	105
**NWR**	**64**		**55**	83
**VIQ**				
**PIQ**			89	125
No sample available for Mother or Sib				

SUPPLEMENTARY FIGURE S4 - Probands with multiple novel or rare variants in one gene

We focus here on potential compound heterozygotes only. Variants are shown in map order with '-' denoting homozygote wildtype and '+' denoting variant carrier. Pedigrees in which the variants were inherited from opposite parents and co-segregated with disorder in the children are shown in Figure 3 of the main manuscript.

chr2:32689842&chr2:32740353, Probands 26&42 – *BIRC6*, P1736L&A3622V

Both variants inherited from single parent

chr2:167089942&chr2:167094638, Proband 2 – *SCN9A*, L1267V&N1245S

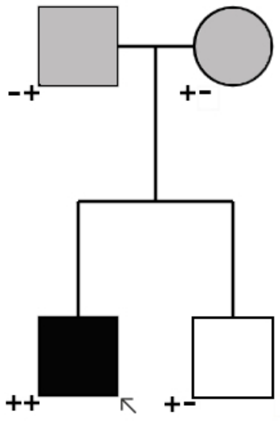

	Father	Mother	Proband	Sib
**chr2:167089942**	GG	G**C**	G**C**	G**C**
**chr2:167094638**	T**C**	TT	T**C**	TT
**ELS**			**62**	112
**RLS**			95	101
**NWR**	**61**	**74**	**65**	96
**VIQ**				
**PIQ**			108	110

chr3:58104626&chr3:58110119, Proband 40 – *FLNB*, G925C&G1262A

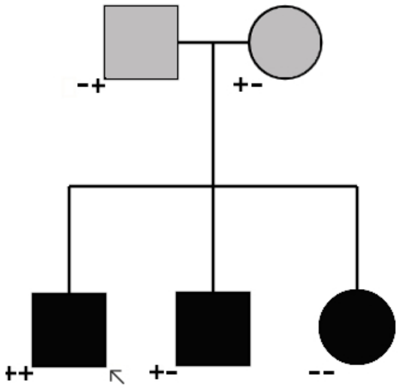

	Father	Mother	Proband	Sib1	Sib2
chr3:58104626	GG	G**T**	G**T**	G**T**	GG
chr3:58110119	G**C**	GG	G**C**	GG	GG
**ELS**			**72**	80	**65**
**RLS**			99	**74**	**69**
**NWR**	88	78	85	89	92
**VIQ**			99	101	93
**PIQ**			104	121	**67**
Both parents self-report a history of speech and language difficulties					

chr11:6190710&chr11:6190828, Proband 19 – *OR52B2*, V283M&C243X

Pedigree shown in Figure 3

SUPPLEMENTARY FIGURE S4 (cont) - Probands with multiple novel or rare variants in one gene

chr11:92086828&chr11:92624235, Proband 7 – *FAT3*, L517S&L4544F

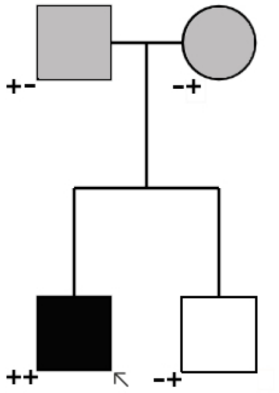

	Father	Mother	Proband	Sib
chr11:92086828	T**C**	TT	T**C**	TT
chr11:92624235	CC	C**T**	C**T**	C**T**
**ELS**			**72**	86
**RLS**			**72**	93
**NWR**	104	91	92	103
**VIQ**				
**PIQ**			119	119

chr12:49418717&chr12:49432365, Proband 12 – *KMT2D*, R5266H&A2925V
Pedigree shown in Figure 3

chr13:109613971&chr13:109617108, Proband 28 – *MYO16*, A708T&splice acceptor lost

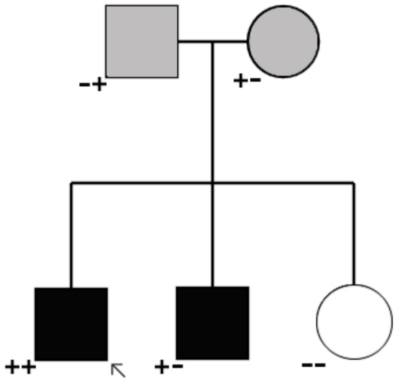

	Father	Mother	Proband	Sib1	Sib2
chr13:109613971	G**A**	GG	G**A**	G**A**	GG
chr13:109617108	GG	G**A**	G**A**	GG	GG
**ELS**			**72**	**73**	84
**RLS**			**76**	85	87
**NWR**	94	91	97	105	105
**VIQ**					
**PIQ**				119	
Father self-reports a history of speech and language difficulties					

chr14:58924684&chr14:59014632, Proband 19 – KIAA086, L577I&G1625R
Pedigree shown in Figure 3

chr15:42977116& chr15:42981101& chr15:42982237, Proband 12 – *STARD9*, C1114R&P2442R&V2821L
Pedigree shown in Figure 3

chr15:42977810& chr15:42978141& chr15:42981101& chr15:42982237, Proband 19 – STARD9, I1345S&E1455D&P2442R&V2821L
Pedigree shown in Figure 3

SUPPLEMENTARY FIGURE S4 (cont) - Probands with multiple novel or rare variants in one gene

chr16:23635348&chr16:23641275, Proband 13 – *PALB2*, L939W&T734S

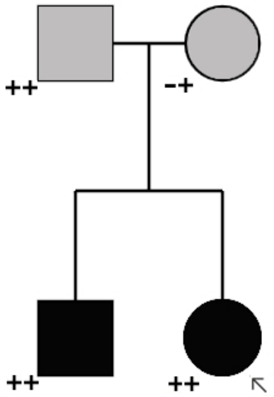

	Father	Mother	Sib	Proband
chr16:23635348	A**C**	AA	A**C**	A**C**
chr16:23641275	T**A**	T**A**	T**A**	T**A**
**ELS**			**64**	**73**
**RLS**			**65**	85
**NWR**	91	**55**		77
**VIQ**				
**PIQ**			108	89

chr17:34861135&chr17:34871802, Proband 25 – *MYO19*, splice donor lost &Y149C
Both variants inherited from single parent
